# The Prevalence of Mental Disorders among the Homeless in Western Countries: Systematic Review and Meta-Regression Analysis

**DOI:** 10.1371/journal.pmed.0050225

**Published:** 2008-12-02

**Authors:** Seena Fazel, Vivek Khosla, Helen Doll, John Geddes

**Affiliations:** 1 Department of Psychiatry, University of Oxford, Warneford Hospital, Oxford, United Kingdom; 2 Oxford Clinic, Oxfordshire and Buckinghamshire Mental Health Foundation NHS Trust, Oxford, United Kingdom; 3 Department of Public Health, University of Oxford, Old Road Campus, Oxford, United Kingdom; University of Queensland, Australia

## Abstract

**Background:**

There are well over a million homeless people in Western Europe and North America, but reliable estimates of the prevalence of major mental disorders among this population are lacking. We undertook a systematic review of surveys of such disorders in homeless people.

**Methods and Findings:**

We searched for surveys of the prevalence of psychotic illness, major depression, alcohol and drug dependence, and personality disorder that were based on interviews of samples of unselected homeless people. We searched bibliographic indexes, scanned reference lists, and corresponded with authors. We explored potential sources of any observed heterogeneity in the estimates by meta-regression analysis, including geographical region, sample size, and diagnostic method. Twenty-nine eligible surveys provided estimates obtained from 5,684 homeless individuals from seven countries. Substantial heterogeneity was observed in prevalence estimates for mental disorders among the studies (all Cochran's χ^2^ significant at *p* < 0.001 and all *I* ^2^ > 85%). The most common mental disorders were alcohol dependence, which ranged from 8.1% to 58.5%, and drug dependence, which ranged from 4.5% to 54.2%. For psychotic illness, the prevalence ranged from 2.8% to 42.3%, with similar findings for major depression. The prevalence of alcohol dependence was found to have increased over recent decades.

**Conclusions:**

Homeless people in Western countries are substantially more likely to have alcohol and drug dependence than the age-matched general population in those countries, and the prevalences of psychotic illnesses and personality disorders are higher. Models of psychiatric and social care that can best meet these mental health needs requires further investigation.

## Abstract 2

Seena Fazel and colleagues show, through a systematic review and meta-regression analysis, that homeless people in Western countries have a higher prevalence of alcohol and drug dependence and mental disorders.

## Editors' Summary

**Background.:**

In 2007, it was estimated that there were more than 1 million homeless people worldwide. The true magnitude of the problem is difficult to estimate with no internationally agreed definition for homelessness and with the different approaches taken by countries and organizations in counting homeless people.

What we do know is that this is a diverse group of people who have poorer physical and mental health than the general population, leading to premature death. We also know that addressing barriers to health care and behavioral interventions for alcohol and drug dependence and mental health problems in this population can lead to lasting health gains.

**Why Was This Study Done?:**

Health care for the homeless is a major public health challenge. Public policy and health service development depend on reliable estimates of the prevalence (how common a particular characteristic, e.g., a disease, is in a specific group of people or a specific population) of illnesses. By using statistical methods, the researchers aimed to provide a quantitative synthesis of the available evidence on mental health problems in this population and explore reasons for the differences in reported prevalence rates of serious mental disorders between studies, neither which have been done previously.

**What Did the Researchers Do and Find?:**

The researchers systematically searched for surveys that estimated the prevalence of mental disorders in homeless people. Their final sample of 29 studies included a total of 5,684 homeless individuals based in the US, UK, mainland Europe, and Australia. Their main finding was that the prevalences of serious mental disorders were raised compared with expected rates in the general population, and many orders of magnitude higher than age-matched community estimates for psychosis, alcohol dependence, and drug dependence. In addition, the analysis found that alcohol and drug dependence is the most common mental disorder in the homeless (compared to psychosis, depression, and personality disorder). Also, the prevalence estimates of psychosis were found to be as high as those for depression. This latter finding contrasts with community estimates and other “at risk” populations such as prisoners and refugees, where depression is more common. The authors found substantial variation in the prevalence rates for these various disorders, and demonstrated that participation rates were associated with these variations for psychosis, depression, and personality disorder and that studies conducted more recently reported higher rates of alcohol dependence.

**What Do These Findings Mean?:**

This review raises a number of implications for health services for the homeless and research for this population. First, traditional models of service delivery, which focus on those with severe mental illness, may not meet the mental health needs of most homeless people who suffer from alcohol and drug dependence and personality disorder. Second, an integrated approach to treatment may be beneficial and should take into account mental health, alcohol and drug abuse, welfare, and housing needs. Finally, future research should include studies that follow a group over time to help us better understand the risks and pathways into (and out of) homelessness, particularly in non-Western populations where there appears to be a paucity of information.

**Additional Information.:**

Please access these Web sites via the online version of this summary at http://dx.doi.org/10.1371/journal.pmed.0050225.

- This study is further discussed in a *PLoS Medicine* Perspective by Helen Herrman
- “How can health care systems effectively deal with the major health care needs of homeless people?” is a WHO initiative aimed at tackling the health care needs of homeless people
- FEANTSA, the European Federation of National Organizations Working with the Homeless, is an umbrella of not-for-profit organizations that participate in or contribute to the fight against homelessness in Europe
- The National Alliance to End Homelessness is a nonpartisan, mission-driven organization committed to preventing and ending homelessness in the US
- Information and good practice solutions for the homelessness service sector in Australia can be found on the National Homelessness Information Clearinghouse Web site
- Homeless Link is the national membership organization for frontline homelessness agencies in England with a mission to catalyze an end to homelessness
- Homeless Man Speaks provides an “on-the-street” perspective

## Introduction

Around 380,000 individuals in the United Kingdom [1] and 740,000 individuals in the United States [2] are reported to be homeless at any given time. Although most live in sheltered accommodation such as emergency hostels, bed and breakfasts, squats, or other temporary accommodation, a recent US report has estimated that 44% are unsheltered, equivalent to over 300,000 people living on the streets [2].

Many studies have reported high prevalences of various health problems among the homeless. Serious physical morbidity, such as tuberculosis and HIV [3], contributes to an increased age-standardised mortality rate of three to four times that in the general population [4,5]. In addition, a number of surveys have found higher rates of serious mental disorders in homeless individuals, but these investigations show at least 10-fold variations in prevalence. Diagnoses of psychosis range from 2% [6] to 31% [7], depression from 4% [8] to 41% [9], and personality disorder from 3% [9] to 71% [10]. It is has been argued that sample selection, case definition, and diagnostic criteria contribute to this heterogeneity [11], although this hypothesis does not appear to have been formally tested. Furthermore, the closure of large psychiatric institutions [12], the shortage of low-cost housing [13], and a lack of community-based supports and services [14] over the past few decades is thought to have contributed to increasing homelessness among people with mental illness, with resulting increased levels of psychiatric morbidity amongst homeless people [8,15]. With the continued reduction in the numbers of inpatient psychiatric beds, the number and proportion of mentally disordered homeless persons is anticipated to increase further [16,17].

Apart from contributing to increased rates of mortality, including from suicide [5,18] and drug abuse [4], the presence of serious mental disorders in the homeless is likely to contribute to increased rates of violent victimization [19], criminality [20–22], and longer periods of homelessness [23]. The provision of good mental health care would therefore reduce psychiatric morbidity and have other public health benefits.

More reliable estimates of the prevalence of serious mental disorders in the homeless should help inform public policy and development of psychiatric services, particularly in urban centres. The most recent review of homeless and mental disorders from 2001 was descriptive and did not attempt a quantitative synthesis of the evidence, or explore the heterogeneity between studies [3]. We report a systematic review and meta-regression analysis of psychiatric surveys of homeless populations in Western countries.

## Methods

We searched for surveys that estimated the prevalence of psychotic illness, major depression, personality disorder, alcohol dependence, and substance dependence in homeless people, published between January 1966 and December 2007. We searched computer-based literature indexes (EMBASE, MEDLINE, PsycINFO), scanned relevant reference lists, searched relevant journals by hand, and corresponded with authors. For the database search, we used combinations of keywords relating to psychiatric illnesses (e.g., mental*, psych*, depress*, substance/drug*/alcohol* abuse/dependence, personality) and being homeless (e.g., homeless*, roofless, shelter*). Non-English articles were translated. MOOSE guidelines were followed (Text S1).

For inclusion into the systematic review, the studies had to meet the following criteria: (1) A clear definition of homelessness was included; (2) standardized diagnostic criteria for psychiatric disorders using the International Classification of Diseases: Classification of Mental and Behavioural Disorders (ICD) or Diagnostic and Statistical Manual of Mental Disorders (DSM) were used; (3) psychiatric diagnosis was made by clinical examination or interviews using validated diagnostic instruments; (4) prevalence rates for psychiatric disorders within the previous six months were included, except for personality disorder where lifetime diagnoses were used; (5) study location was in North America, Western Europe, Australia, and New Zealand.

Surveys with less than a 50% response rate were excluded, as were surveys on selected populations (for example, a sample of homeless people referred to a psychiatric outpatient clinic or single mothers [24]) or where diagnosis of psychiatric disorders was not obtained by direct interviews (for example, by self report or case note review) or was only reported as 12-mo or lifetime diagnoses [16,25–29]. Studies that selected solely elderly or juvenile people were excluded [30,31]. All the included reports were based on interviews with individuals. We identified one study that interviewed families, but this was excluded as it was based on a selected sample [32].

Information on geographical location; year of interview; definition of homelessness; method of sample selection; sample size; average age; diagnostic instrument; diagnostic criteria, type of interviewer, participation rate; and numbers diagnosed with psychotic illness, major depression, personality disorder, alcohol dependence, and drug dependence was extracted independently from every eligible study. If required, further clarifications were sought by correspondence with authors of relevant studies.

Prevalence estimates were calculated using the variance stabilising double arcsine transformation [33], because of the use of the inverse variance weight in fixed-effects meta-analysis is suboptimal when dealing with binary data with low prevalences. In addition, the transformed prevalences are weighted very slightly towards 50% and studies with zero prevalence can thus be included in the analysis. Confidence intervals (CIs) around these estimates were calculated using the Wilson method [34] since the asymptotic method produces intervals which can extend below zero [35]. Heterogeneity among studies was estimated using Cochran's *Q* (reported with a χ^2^-value and *p*-value) and the *I* ^2^ statistic, the latter describing the percentage of variation across studies that is due to heterogeneity rather than chance [36,37], and presented with a 95% CI [37]. *I* ^2^, unlike *Q*, does not inherently depend upon the number of studies considered, with values of 25%, 50%, and 75% taken to indicate low, moderate, and high levels of heterogeneity, respectively. Where heterogeneity was high (*I* ^2^ > 75%), random effects models were used for summary statistics [36]. In situations with high between-study heterogeneity, the use of random effects models (where the individual study weight is the sum of the weight used in a fixed effects model and the between-study variability) produces study weights that primarily reflect the between-study variation and thus provide close to equal weighting. The use of the arcsine-transformed prevalence estimates consequently had little material difference on the value of the overall random effects estimates, which were themselves found to be notably different (closer to 50%) from the fixed effects estimates in which smaller prevalences have smaller standard errors and thus greater weight. Potential sources of heterogeneity were investigated further by arranging groups of studies according to potentially relevant characteristics and by meta-regression analysis. Factors examined both individually and in multiple variable models were instrument (semistructured instrument versus clinical examination only), interviewer (conducted by mental health clinician or not), period (decade of study: 1970s, 1980s, 1990s, and 2000s), study size (both a continuous variable and as a categorical variable in increments of *n* = 50 and *n* = 100, and also as *n* < 200 versus *n* ≥ 200), sex (as appropriate, e.g., male versus female; mixed versus male versus female), geographical region (as appropriate, e.g., mainland Europe versus rest of Western countries), and participation rates (classified into those >85% and those ≤85%). Because of low prevalence and small sample size for some studies, only those factors significant (*p* < 0.10) individually were entered into a multiple regression model to avoid model instability. The regression coefficients for each study characteristic on individual analysis were provided to enable comparison across diagnoses. All analyses were done in STATA statistical software package, version 10 (Statacorp, 2007) using the commands propcii (to calculate Wilson CIs), metan (for random effects meta-analysis, specifying either two or three variables: double arcsine transformed prevalence and its variance, or double arcsine transformed prevalence and Wilson CIs), and metareg (for meta-regression).

## Results

The final sample consisted of 29 studies published between 1979 and 2005 (Table 1) [6–10,38–59]. The studies included a total of 5,684 homeless individuals. Eleven reports reported data on men (*n* = 1,827) [7,38,40–43,45,47–49,60], 14 included mixed gender samples (*n* = 3,381) [6,8,39,44,50–54,61], and four investigated women (*n* = 476) [9,10,40,55]. In the surveys with mixed samples, 82% of the individuals were men (weighted average). The weighted average age of men (reported in eight reports) and women (from four reports) was 41.2 and 33.8 y, respectively. Average age of the mixed samples was 40.1 y (from ten studies). Ten studies were published before 1990 [6–8,10,39,40,42,45,46,49]. Ten reports were from the US (*n* = 2,019) [6,8,10,38,39,42,49,51,52,55], eight from the UK (*n* = 1645) [7,44,45,53,56–58,61], six from Germany (*n* = 624) [9,41,47,48,59,60], two from Australia (*n* = 473) [40,46], and one each from France (*n* = 715) [50], The Netherlands (*n* = 150) [43], and Greece (*n* = 58) [54].

**Table 1:**
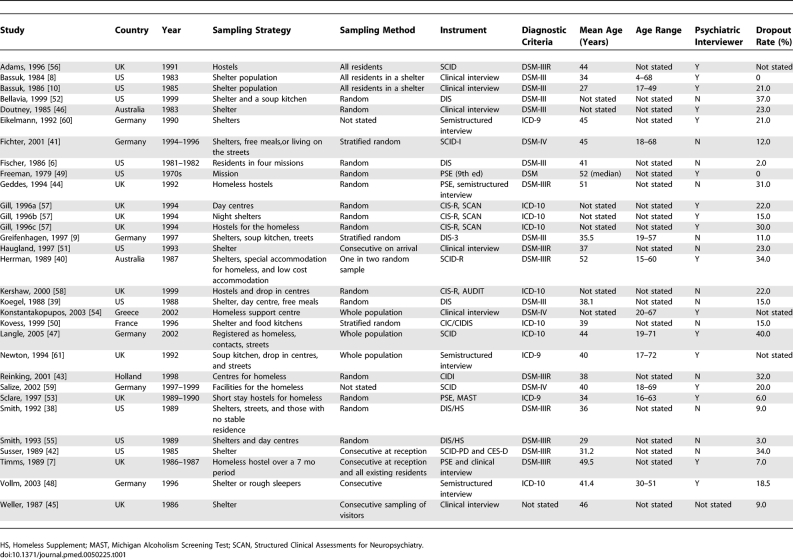
Details of Studies on Prevalence of Mental Disorders in the Homeless

Different sampling strategies (random methods, consecutive sampling or total sampling) were used in surveys, apart from two reports that did not provide information on sampling method [59,60]. Of the 5,684 homeless individuals, 952 were selected primarily from shelters for the homeless, 583 from hostels for the homeless, and the rest from a mixture of settings including day centres, soup kitchens, missions, streets, and shelters. Eight studies reported response rate of above 90% [6–8,38,45,49,53,55], the others were mostly above 70%. Three reports did not provide response rates [54,56,61], a further one did not cite the response rate for part of the sample [40], and five surveys reported response rates between 60% and 70% [42–44,47,52].

In six surveys, diagnosis of psychotic illness, major depression, personality disorder, alcohol dependence, and drug dependence was made by clinical examination without the use of diagnostic instruments (*n* = 600) [7,8,10,51,54]. Validated semistructured diagnostic instruments used included: Diagnostic Interview Schedule (DIS) (*n* = 1,455) [6,9,38,39,52,55]; Clinical Interview Schedule, Revised (CIS-R) (*n* = 1,090) [57,58]; Structured Clinical Interview for Diagnostic and Statistical Manual (SCID) (*n* = 1,081) [40,41,47,56,59]; Present State Examination (PSE) (*n* = 394) [44,45,49,53], and the Composite International Diagnostic Interview (CIDI) (*n* = 724) [43,48,50]. Three other studies used clinical semistructured interviews [48,60,61].

### Psychotic Illness

**Figure 1:**
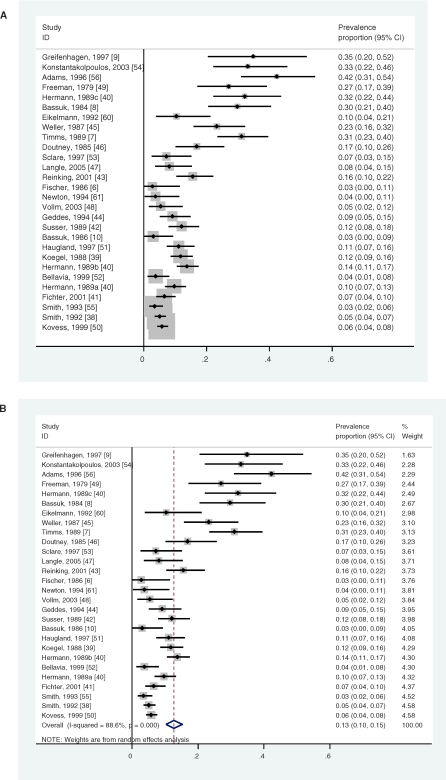
Prevalence of Psychosis in Homeless Persons (A) Studies are ordered by increasing study weight in a fixed effects model without calculation of an overall estimate. (B) Studies are ordered by increasing study weight and show the overall estimate calculated from random effects meta-analysis.

**Figure 2:**
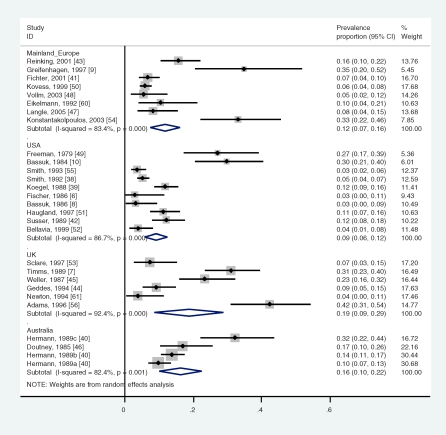
Prevalence of Psychosis in Homeless Persons by Country Group

**Figure 3:**
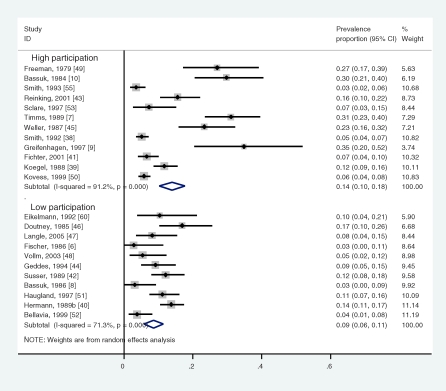
Prevalence of Psychosis in Homeless Persons by Participation Rate

Psychotic illness was reported in 28 surveys with a random effects pooled prevalence of 12.7% (95% CI 10.2%–15.2%) [6–10,38–56,60,61]. Estimates ranged from 2.8% to 42.3% with substantial heterogeneity among the estimates (χ^2^ = 237.7, *p* < 0.001, *I* ^2^ = 88.6%, 95% CI 84.8%–91.5%). Higher prevalences were found in smaller studies (Figure 1). We further explored study region, grouping studies according to whether they were based in mainland Europe, UK, US, or Australasia (Figure 2). In individual variable meta-regression analyses, surveys in which the interviewer was a mental health clinician (versus a lay interviewer) had higher prevalences of psychosis (β = 0.08, standard error [se(β)] = 0.04, *p* = 0.042), where the participation rates were lower (<85%) had lower prevalences (β = −0.06, se[β] = 0.03, *p* = 0.071) (Figure 3), and where the study size was 200 or more participants (versus fewer than 200) had lower prevalences (β = −0.08, se[β] = 0.04, *p* = 0.055) (Table 2). In a meta-regression model including these three characteristics, only the participation rate remained significant: lower participation rates were associated with lower prevalences (β = −0.08, se[β] = 0.03, *p* = 0.015).

**Table 2:**
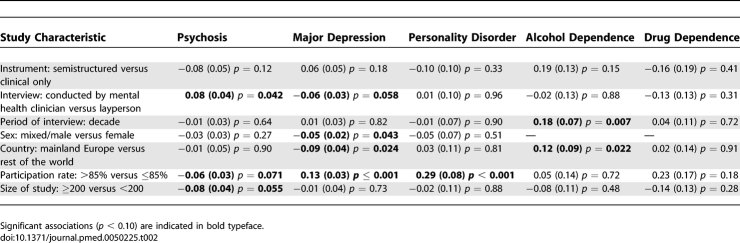
Results of Individual Variable Meta-Regression Models for Each Diagnosis Showing Values of β, se(β), and the Significance of β for Each Study Characteristic

### Major Depression

**Figure 4:**
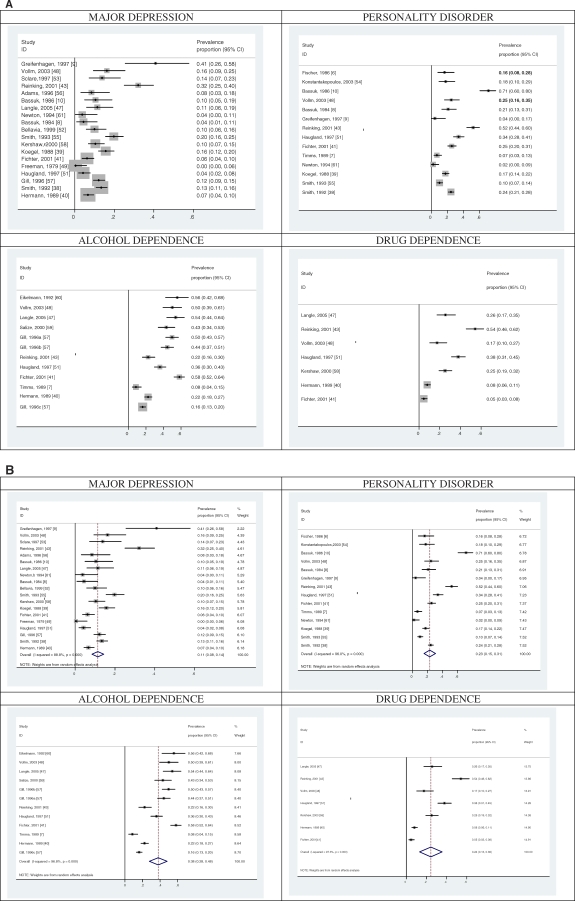
Prevalence of Major Depression, Personality Disorder, Alcohol Dependence, and Drug Dependence in Homeless People (A) Studies are ordered by increasing study weight in a fixed effects model without calculation of an overall estimate. (B) Studies are ordered by increasing study weight and show the overall estimate calculated from random effects meta-analysis.

We identified 19 surveys that reported major depression with a random effects pooled prevalence estimate of 11.4% (95% CI 8.4%–14.4%) (Figure 4) [8–10,38–41,43,47–49,51–53,55–58,61]. The prevalence estimates ranged from 0.0% to 40.9% and there was substantial heterogeneity among those estimates (χ^2^ = 160.6, *p* < 0.001, *I* ^2^ = 88.8%, 95% CI 84.0%–92.2%). Four factors were associated with this heterogeneity on meta-regression: the interview being conducted by a mental health clinician, where there were lower prevalences (β = −0.06, se[β] = 0.04, *p* = 0.058); the study being conducted in mainland Europe compared with the rest of the world (β = −0.09, se[β] = 0.04, *p* = 0.024); sex (coded as female, male, mixed), with men and mixed samples having progressively lower prevalences than women (β = −0.05, se[β] = 0.02, *p* = 0.043); and participation rate, with lower participation rates being associated with higher prevalences (β = 0.13, se[β] = 0.03, *p* < 0.001) (Table 2). In a meta-regression model including these four characteristics, only the participation rate remained significant: lower participation rates were associated with higher prevalences (β = 0.11, se[β] = 0.03, *p* = 0.002).

### Personality Disorder

We identified 14 surveys that reported personality disorder diagnoses in 2,413 homeless persons (Figure 4) [6–10,38,39,41,48,51,54,55,61]. The prevalence estimates ranged from 2.2% to 71.0%. The random effects pooled prevalence estimate was 23.1% (95% CI 15.5%–30.8%) (χ^2^ = 327.5, *p* < 0.001, *I* ^2^ = 96.0%, 95% CI 94.6%–97.1%), with only one of the sample characteristics being significantly associated with this substantial heterogeneity on individual variable analysis: lower participation rates were associated with higher prevalences (β = 0.29, se[β] = 0.08, *p* < 0.001) (Table 2).

### Alcohol Dependence

**Figure 5:**
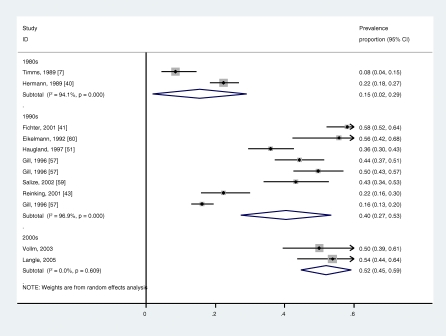
Prevalence of Alcohol Dependence in Homeless Persons by Decade

We identified ten surveys that reported alcohol dependence in 1,791 homeless men (Figure 4) [7,40,41,43,47,48,51,57,59,60]. The prevalence estimates ranged from 8.5% to 58.1%. The random effects pooled prevalence estimate was 37.9% (95% CI 27.8%–48.0%) (χ^2^ = 347.2, *p* < 0.001, *I* ^2^ = 96.8%, 95% CI 95.7%–97.7%). Two factors were associated with this heterogeneity on individual but not multiple variable analysis. First, the more recent the study (as analysed by decade), the higher the prevalence of alcohol dependence (β = 0.18, se[β] = 0.07, *p* = 0.007, Figure 5). Second, surveys conducted in mainland Europe had higher prevalences of alcohol dependence (β = 0.21, se[β] = 0.09, *p* = 0.022) (Table 2). We did not find any investigations of alcohol dependence in homeless women.

### Drug Dependence

We identified seven surveys in men that reported drug dependence (Figure 4) [40,41,43,47,48,51,58]. The prevalence estimates ranged from 4.7% to 54.2%. Random effects pooled prevalence estimate was 24.4% (95% CI 13.2%–35.6%) (χ^2^ = 221.1, *p* < 0.001, *I* ^2^ = 97.3%, 95% CI 95.9%–98.2%), with none of the sample characteristics being significantly associated with this heterogeneity on individual variable analysis (Table 2). We found one report on drug dependence in homeless women (*n* = 33) with a prevalence of 24.2% [58].

## Conclusion

This systematic review of serious mental disorders in homeless persons identified 29 surveys including 5,684 individuals. There are three main findings. First, the most common mental disorders appeared to be alcohol and drug dependence, with random effects pooled prevalence estimates of 37.9% (95% CI 27.8%–48.0%) and 24.4% (95% CI 13.2%–35.6%), respectively. Second, the prevalence estimates for psychosis were at least as high as those for depression, a finding in marked contrast from community estimates of these conditions [62,63], and in other at-risk groups such as prisoners [64] and refugees [65], in whom depression is more common. Third, although high prevalences were reported for serious mental disorders, their substantial heterogeneity suggests that service planning should not rely on our summary estimates but commission local surveys of morbidity to quantify mental health needs.

The substantial heterogeneity between the studies included in the review was not unexpected. Some of the variations between studies could be not explained by crude study characteristics (such as sex, study size, or geographic region), and this suggests that local factors such as provision of mental health and social services are likely to be important [66]. However, certain other study characteristics were associated with heterogeneity on meta-regression. These included, first, lower participation in surveys were associated with lower prevalences of psychosis. Second, interviewers with clinical training were more likely to reporting lower prevalences of depression. Third, a higher prevalence of alcohol dependence was found in studies in more recent decades, possibly reflecting the increasing relative affordability of alcohol [67,68].

The prevalence of psychosis ranged between 3% and 42%, which is substantially higher than community estimates, which typically report 1%–2% when individuals are surveyed using diagnostic instruments [69], and up to 0.8% when only schizophrenia is reported [70]. We found no increase in the prevalence of psychosis by study period (in decades), in contrast with the views of some commentators [15,16]. The finding on meta-regression that the proportion of the individuals who participated was significantly associated with these prevalences suggests that researchers in the field need to interpret their findings in light of response rates. Reasons for nonparticipation in research may be related to severe mental illness, and investigators should consider gathering information from other sources to estimate the degree of underestimation or attempt to reinterview those who did not participate initially.

Although the proportionate excess for psychosis was greater than for the other mental disorders, this review found that the main mental health problem for homeless persons is alcohol dependence, which ranged from 8% to 58%. On average, this is many orders of magnitude higher in men and even higher in women compared with community surveys [62,63]. The range for drug dependence were 5%–54%, a proportionate excess higher in women than men compared with community surveys [62,63]. The increased prevalence for drug dependence in both sexes may contribute to the reported increased crime rates in the homeless [20,21,22], as substance abuse and dependence is an important risk factor, either alone [71] or comorbid with psychosis [21].

The prevalence of depression in this review, with a pooled prevalence estimate of 11.4% (95% CI 8.4%–14.4%), range at 0% to 41%, was lower than expected. Many estimates included in the review were similar to community estimates of depression in women (which range from 7% to 11% for 1-y estimates), and only slightly higher than general population rates for men [62,72]. While levels of comorbidity of depression are likely to be underreported in homeless persons with psychosis or drug dependence, these estimates suggest that the reported high rates of suicide in homeless people [5,18] may not be mediated through depressive illness, and that other risk factors, such alcohol dependence, may be more relevant [73]. The finding that clinically trained interviewers were associated with lower prevalence estimates is consistent with a systematic review of major depressive disorder in prisoners [64], and suggests that studies of the diagnostic validity of depression using different types of interviewer should be researched. In terms of the prevalence of personality disorder in the homeless, we found that the estimates varied widely (range 2.2%–71.0%), and all but one reported rates higher than the 4.4% found in a recent community survey [74]. The presence of personality disorder is associated with poorer outcomes for treatment of depression [75] and increased risk of deliberate self-harm [76].

One of the findings of this review is that future research in homeless populations should provide clear definitions of the sample interviewed and breakdowns by type of homelessness category (whether it be roofless or temporary accommodation). Although we identified and excluded from the review one study from a non-Western population (South Korea) [77], it highlighted the need for research in non-Western countries. Longitudinal studies of cohorts of the mentally ill would help clarify the risk for, temporal sequence of, and pathways into homelessness, and identify risk factors and those associated with desistence for homelessness in such populations.

A number of implications for health services for the homeless arise from this review. Integrating treatment for mental illness, substance dependence, and housing interventions should be considered for many homeless persons [78,79], and hospital admission may be required for treatment for a number with psychotic illness. Local surveys are needed to inform service needs. However, traditional models of service delivery in Western countries, which focus on those with severe mental illness, may not meet the mental health needs of most homeless people who suffer from substance dependence and personality disorder. Even when mentally ill homeless persons receive adequate mental health services, a range of unmet welfare and housing needs may remain, implying that normal community mental health service provision is usually insufficient [80]. With many millions of individuals being homeless in Western countries, current mental health provision may need review, and models of psychiatric and social care that can best meet the burden of mental illness will need further investigation.

## Supporting Information

Text S1 MOOSE Checklist (63 KB DOC)

## Abbreviations

CI: confidence interval
CIDI: Composite International Diagnostic Interview
CIS-R: Clinical Interview Schedule, Revised
DIS: Diagnostic Interview Schedule
DSM: Diagnostic and Statistical Manual of Mental Disorders
ICD: International Classification of Diseases: Classification of Mental and Behavioural Disorders
PSE: Present State Examination
SCID: Structured Clinical Interview for Diagnostic and Statistical Manual
se: standard error
