# Supplementary material for: The Prevalence of Mental Disorders among the Homeless in Western Countries: Systematic Review and Meta-Regression Analysis
Source: PLoS Med. 2008 Dec 2;5(12):e225. doi: 10.1371/journal.pmed.0050225 (PMC2592351; doi:10.1371/journal.pmed.0050225)
Supplement: Supplementary file 1 [file pmed.0050225.sd001.doc]

**Reporting Checklist for Meta-analyses of Observational Studies (MOOSE) for PLoS Medicine**

|  | Reported? | MS page | Art. page |
| --- | --- | --- | --- |
| Reporting of background should include: |  |  |  |
| Problem definition | Yes | 4, 5 |  |
| Hypothesis statement | Yes | 4, 5 |  |
| Description of study outcome(s) | Yes | 5 |  |
| Type of exposure or intervention used | Yes | 4, 5 |  |
| Type of study designs used | Yes | 6 |  |
| Study population | Yes | 4 |  |
| Reporting of search strategy should include: |  |  |  |
| Qualifications of searchers (eg, librarians and investigators) | Yes | 1 |  |
| Search strategy, including time period included in the synthesis and keywords | Yes | 6 |  |
| Effort to include all available studies, including contact with authors | Yes | 6 |  |
| Databases and registries searched | Yes | 6 |  |
| Search software used, name and version, including special features used (eg, explosion) | No |  |  |
| Use of hand searching (eg, reference lists of obtained articles) | Yes | 6 |  |
| List of citations located and those excluded, including justification | Yes | 6, 7 |  |
| Method of addressing articles published in languages other than English | Yes | 6 |  |
| Method of handling abstracts and unpublished studies | No |  |  |
| Description of any contact with authors | Yes | 6 |  |
| Reporting of methods should include: |  |  |  |
| Description of relevance or appropriateness of studies assembled for assessing the hypothesis to be tested | Yes | 6 |  |
| Rationale for the selection and coding of data (eg, sound clinical principles or convenience) | Yes | 7 |  |
| Rationale for the selection and coding of data (eg, sound clinical principles or convenience) | Yes/No |  |  |
| Documentation of how data were classified and coded (eg, multiple raters, blinding, and interrater reliability) | Yes | 7 |  |
| Assessment of confounding (eg, comparability of cases and controls in studies where appropriate) | No |  |  |
| Assessment of study quality, including blinding of quality assessors; stratification or regression on possible predictors of study results | Yes | 7, 8 |  |
| Assessment of heterogeneity | Yes | 7, 8 |  |
| Description of statistical methods (eg, complete description of fixed or random effects models, justification of whether the chosen models account for predictors of study results, dose-response models, or cumulative meta-analysis) in sufficient detail to be replicated | Yes | 7,8 |  |
| Provision of appropriate tables and graphics | Yes | 23 |  |
| Reporting of results should include: |  |  |  |
| Graphic summarizing individual study estimates and overall estimate | Yes | Fig 1 & 4 |  |
| Table giving descriptive information for each study included | Yes | Table 1 |  |
| Results of sensitivity testing ( eg, subgroup analysis) | Yes | 11-14, Fig 2,3 5 |  |
| Indication of statistical uncertainty of findings | Yes | 11-14 |  |
| Reporting of discussion should include: |  |  |  |
| Quantitative assessment of bias (eg, publication bias) | Yes | 15 |  |
| Justification of exclusion (eg, exclusion of non-English-language citations) | Yes | 17 |  |
| Assessment of quality of included studies | Yes | 15 |  |
| Reporting of conclusions should include: |  |  |  |
| Consideration of alternative explanations for observed results | Yes | 15 |  |
| Generalization of the conclusions (ie, appropriate for the data presented and within the domain of the literature review) | Yes | 15-18 |  |
| Guidelines for future research | Yes | 17 |  |
| Disclosure of funding source | Yes | 1 |  |

- Stroup DF, Berlin JA, Morton SC, Olkin I, Williamson GD, Rennie D, Moher D, Becker BJ, Sipe TA, Thacker SB. Meta-analysis of observational studies in epidemiology: a proposal for reporting. Meta-analysis Of Observational Studies in Epidemiology (MOOSE) group. JAMA 2000; 283(15):2008-2012.
